# Deaths due to Unknown Foodborne Agents

**DOI:** 10.3201/eid1009.030403

**Published:** 2004-09

**Authors:** Paul D. Frenzen

**Affiliations:** * U.S. Department of Agriculture, Washington, D.C., USA

**Keywords:** mortality, cause of death, infection, gastroenteritis, United States, perspective

## Abstract

This study reviews the available evidence on unknown pathogenic agents transmitted in food and examines the methods that have been used to estimate that such agents cause 3,400 deaths per year in the United States. The estimate of deaths was derived from hospital discharge and death certificate data on deaths attributed to gastroenteritis of unknown cause. Fatal illnesses due to unknown foodborne agents do not always involve gastroenteritis, and gastroenteritis may not be accurately diagnosed or reported on hospital charts or death certificates. The death estimate consequently omitted deaths from unknown foodborne agents that do not cause gastroenteritis and likely overstated the number of deaths from agents that cause gastroenteritis. Although the number of deaths from unknown foodborne agents is uncertain, the possible economic cost of these deaths is so large that increased efforts to identify the causal agents are warranted.

## Abstract 2

The number of U.S. deaths by unknown foodborne agents warrants additional efforts to identify causal agent.

Estimates of foodborne disease deaths are subject to uncertainty because the number of deaths caused by unidentified pathogenic agents in the food supply is unknown. These agents may include bacteria, viruses, parasites, toxins, viruslike agents, or prions. In their influential study of foodborne disease in the United States, Mead et al. (*1*) estimated that unknown foodborne agents caused 3,400 deaths per year, or 65% of the estimated 5,200 annual deaths from foodborne disease. The Mead study indicates that unknown foodborne agents are an important cause of premature death, with an annual toll comparable to that from accidental fires (3,300 deaths) and drownings (3,300 deaths) (*2*).

Unknown foodborne agents are unlikely to be reported as a cause of death. The Mead study used hospital discharge and death certificate data on deaths attributed to gastroenteritis of unknown cause to indirectly estimate the number of deaths caused by unknown foodborne agents (*1*). The estimate by the Mead study is the first systematic assessment of deaths from unknown foodborne agents and deserves scrutiny because little is known about such agents. This study examines the available evidence on unknown foodborne agents for the United States and discusses whether the methods used by the Mead study to estimate deaths from unknown foodborne agents are valid and accurate.

## Evidence for Unknown Foodborne Agents

Evidence suggests that unknown agents cause fatal illness. The Unexplained Death and Critical Illness (UNEX) Project detected 35 deaths caused by possible unknown pathogens in previously healthy persons 1–49 years of age in four U.S. surveillance sites from 1995 to 1998 (*3*). Deaths from unknown foodborne agents that occurred during intensive care for illnesses resembling infections would have met the UNEX case definition. The death rate from possible unknown pathogens in the UNEX surveillance sites was equivalent to approximately 200 deaths per year among U.S. residents 1–49 years of age, without adjusting for the superior health of the surveillance population.

The Mead study cited evidence that unknown agents are transmitted in food, including well-documented outbreaks of distinctive foodborne illness caused by unidentified agents and the high proportion of reported foodborne outbreaks with an undetermined cause (*1*). Reported outbreaks probably account for only a small proportion of deaths from unknown foodborne agents because most foodborne outbreaks are never recognized or reported (*4*) and because some deaths from unknown foodborne agents may result from sporadic illness. However, reported outbreaks represent the only direct evidence of deaths attributable to unknown foodborne agents.

Well-documented outbreaks of distinctive foodborne illness caused by unidentified agents are infrequent in the United States and rarely result in death. Only six reports of such outbreaks have appeared in the Morbidity and Mortality Weekly Report (MMWR) since 1990 (*5**–**10*). (A seventh report was excluded because testing for viral agents was incomplete [*11*].) Two of the reports involved agents later identified as seaweed toxin and *Cyclospora cayetanensis*, which was waterborne in the United States but found elsewhere in food (*5**,**7*). A third report concerned Haff disease, which has a case-fatality rate of 1% (*9*). The other reports described three distinctive illnesses, but two of the illnesses did not lead to any deaths and therefore appeared to be nonfatal (*6**,**8**,**10*). Brainerd diarrhea, the best known example of an illness caused by an unknown foodborne agent, also appears to be nonfatal (*12*).

More than two-thirds of the 2,800 foodborne outbreaks reported from 1993 to 1997 had an undetermined cause (*4*). However, most of these outbreaks were probably caused by known pathogens that were not detected, particularly viruses. Recent studies that used molecular diagnostic methods suggest that the causal agents were often members of the *Norovirus* genus (*13*). The 1,900 outbreaks of undetermined cause reported from 1993 to 1997 resulted in only one recorded death (*4*), a pattern consistent with the low case-fatality rate for norovirus infections.

## The Mead Study

Most deaths from unknown foodborne agents are presumably attributed to unknown causes because clinical laboratory tests are unlikely to detect the causal agent. Physicians describe deaths from unknown causes on hospital charts and death certificates by recording the medical conditions that preceded death. Deaths from unknown agents that caused distinctive conditions could be indirectly estimated from the number of unexplained deaths with such conditions. This approach was adopted by the Mead study, which estimated the number of deaths caused by unknown foodborne agents from the number of deaths involving gastroenteritis of unknown cause.

Gastroenteritis is a common clinical feature of enteric infectious diseases. The Mead study obtained data on deaths involving gastroenteritis of unknown cause from two sources, the annual Multiple Cause of Death (multiple cause) files of death certificates (*14*), and the annual National Hospital Discharge Survey (NHDS) of nonfederal short stay and general hospitals (*15*). Causes of death reported on the multiple cause files were coded by using the International Classification of Diseases, ninth revision (ICD-9) from 1979 to 1998, and the 10th revision (ICD-10) after 1998 (*16**,**17*). Diagnoses reported on the NHDS files were coded by using the ICD-9 Clinical Modification (ICD-9-CM) (*18*).

Table 1 lists ICD-9, ICD-9-CM, and ICD-10 codes for gastroenteritis of unknown etiology. In ICD-9, conditions described as "diarrhea" or "gastroenteritis" are coded as 558, together with radiation, toxic, and allergic gastroenteritis. Supplemental injury and external cause codes assigned during cause-of-death coding differentiate radiation and toxic gastroenteritis, as well as gastroenteritis attributable to adverse drug reactions (*19*). In ICD-9-CM, 558 is subdivided into rubrics identifying radiation, toxic, and allergic gastroenteritis, and a new code for diarrhea was added in 1995 (*20*). In ICD-10, the codes for gastroenteritis of unknown etiology distinguish infectious conditions, noninfectious conditions in neonates, and noninfectious conditions at other ages.

**Table 1:** International Classification of Diseases (ICD) codes for gastroenteritis of unknown etiology

ICD revision and code	Title
ICD-9	
Valid for cause of death coding	
009.0	Infectious colitis, enteritis, and gastroenteritis
009.2	Infectious diarrhea
558^a^	Other noninfectious gastroenteritis and colitis
Invalid for cause-of-death coding	
009.1^b^	Colitis, enteritis, and gastroenteritis of presumed infectious origin
009.3^b^	Diarrhea of presumed infectious origin
ICD-9-CM	
009.0	Infectious colitis, enteritis, and gastroenteritis
009.1	Colitis, enteritis, and gastroenteritis of presumed infectious origin
009.2	Infectious diarrhea
009.3	Diarrhea of presumed infectious origin
558.9^c^	Other and unspecified noninfectious gastroenteritis and colitis
787.91^d^	Other symptoms involving digestive system: diarrhea
ICD-10	
A09	Diarrhea and gastroenteritis of infectious origin^e^
K52.9	Noninfective gastroenteritis and colitis, unspecified
P78.3	Noninfective neonatal diarrhea

^a^Includes radiation, toxic, and allergic gastroenteritis. ^b^Coded as 558 on the 1979–1998 Multiple Cause of Death files. ^c^Included allergic gastroenteritis before 2000. ^d^Code introduced in 1995. ^e^Includes conditions that are presumed to be infectious.

In the Mead study, the number of deaths from unknown foodborne agents was estimated in two steps (*1*). First, the annual number of deaths from gastroenteritis of unknown cause was determined by averaging estimates from the 1992–1996 NHDS and multiple cause files. Deaths were classified as gastroenteritis of unknown cause if any cause of death was coded as ICD-9 009 or 558 on the multiple cause files, or if any listed diagnosis was coded as ICD-9-CM 009 or 558.9 on the NHDS files. In the second step, the number of deaths from unknown foodborne agents was determined by assuming that the proportion of foodborne deaths among the 5,000 annual deaths from gastroenteritis of unknown cause was the same as the proportion among deaths from known pathogens that are sometimes or always transmitted through food (67%).

The methods used by the Mead study have several shortcomings. First, the definition of gastroenteritis of unknown cause was imprecise. The definition based on ICD-9 codes included radiation, toxic, and drug-induced gastroenteritis that could be identified and excluded by using the supplemental codes assigned during cause-of-death coding. The definition based on ICD-9-CM codes also included drug-induced gastroenteritis that could be identified by supplemental codes, but excluded gastroenteritis of unknown cause that could be identified by using the new code for diarrhea. Table 2 reports modified ICD-9 and ICD-9-CM definitions of gastroenteritis of unknown cause that avoid these limitations.

**Table 2:** Modified ICD-9 and ICD-9-CM definitions of deaths attributable to gastroenteritis of unknown etiology

ICD-9	ICD-9-CM
Any death with an ICD-9 009.0, 009.2, or 558 code, excluding 1) radiation gastroenteritis deaths, identified by a 558 code plus the supplemental code for an abnormal reaction to a radiological procedure or therapy (E879.2)^a^ 2) toxic gastroenteritis deaths, identified by a 558 code plus any supplemental code for poisoning (909.0, 909.1, 960.0-989.9, E850.0-E869.9, E950.0-E950.9, E980.0-E980.9)^a^ 3) drug-induced diarrhea deaths, identified by a 558 code plus any supplemental code for an adverse drug reaction (E930.0-E949.9), or by a 009.0 or 009.2 code plus any supplemental code for an adverse reaction to an antibiotic (E930.0-E931.0)^a^	Any death with an ICD-9-CM 009.0-009.3, 558.9, or 787.91 code, excluding 1) drug-induced diarrhea deaths, identified by a 558.9 or 787.91 code plus any supplemental code for an adverse drug reaction (E930.0-E949.9), or else by a 009.0-009.3 code plus any supplemental code for an adverse reaction to an antibiotic (E930.0-E931.0)^b^

^a^The supplemental code must be listed on the same line or one line after the code for gastroenteritis of unknown etiology on part I of the death certificate (for conditions resulting in death), or else both codes must be listed together on part II of the death certificate (for other contributing conditions). ^b^Supplemental codes on NHDS files are not explicitly associated with other diagnoses. Therefore, gastroenteritis of unknown etiology was assumed to be drug-induced if any other diagnosis was one of the specified adverse drug reactions.

A second shortcoming of the Mead study was the low reliability of the NHDS estimate of annual deaths from gastroenteritis of unknown cause. Using the modified ICD-9-CM definition of gastroenteritis of unknown cause, the approximate 95% confidence interval (CI) for the NHDS estimate of 6,194 annual deaths from 1992 to 1996 was 1,627–10,761 deaths (*20*). The wide interval was due in part to the small number of such deaths (averaging 45 per year) in the NHDS sample. Death certificate data also show that 29% of deaths from gastroenteritis of unknown cause occurred in nursing homes or other settings outside the hospitals in the NHDS sample (*21*), which indicates that the NHDS estimate was not comprehensive.

The most important shortcomings of the Mead study were the two implicit assumptions about gastroenteritis underlying the estimate of deaths from unknown foodborne agents. The first assumption was that fatal illness attributable to unknown foodborne agents always involved gastroenteritis. The second assumption was that gastroenteritis was accurately diagnosed and reported on hospital charts and death certificates. The two assumptions implied that all deaths from unknown foodborne agents and other unknown agents that cause gastroenteritis (but no other deaths) were attributed to gastroenteritis of unknown cause. Both assumptions are questionable as discussed in the following sections.

### Gastroenteritis Caused by Unknown Foodborne Agents

Fatal illnesses attributable to unknown foodborne agents do not always involve gastroenteritis, contrary to the first implicit assumption by the Mead study. Gastroenteritis is not a clinical feature of the only two illnesses caused by unknown foodborne agents known to be fatal, Haff disease and an illness associated with a batch of Kombucha tea (*8**,**9*). In addition, most deaths from possible unknown pathogens identified by the UNEX project involved cardiac, respiratory, or neurologic syndromes rather than gastroenteritis, although the initial symptoms (which may have included gastroenteritis) were not reported (*3*).

The estimate of deaths from unknown foodborne agents by the Mead study omitted deaths from unknown foodborne agents that do not cause gastroenteritis. The number of such deaths is unknown. However, some known foodborne pathogens cause fatal illnesses that do not involve gastroenteritis. In fact, 51% of the estimated deaths from known foodborne pathogens were attributable to four agents (*Listeria monocytogenes*, *Toxoplasma gondii*, hepatitis A virus, and *Vibrio vulnificus*) that cause fatal illnesses unaccompanied by gastroenteritis (*1*). If unknown foodborne agents were as likely as known foodborne pathogens to cause gastroenteritis, then only one half of the deaths caused by unknown foodborne agents would involve gastroenteritis.

### Gastroenteritis Diagnosis and Reporting

Gastroenteritis is not always accurately diagnosed or reported, contrary to the second implicit assumption by the Mead study. Physicians do not always recognize known causes of gastroenteritis and may not report the specific cause on hospital charts or death certificates even when it has been identified. As a result, some instances of gastroenteritis attributable to known causes were probably coded as gastroenteritis of unknown origin in hospital discharge and mortality databases.

Infectious causes of gastroenteritis may escape detection because of imperfect testing procedures. Many clinical laboratories do not routinely test stool specimens for some enteric pathogens, including *Escherichia coli* O157:H7 (*22*), and do not have reliable tests for other enterohemorrhagic *E. coli* serotypes (*23*). Reliable tests for pathogens like *Campylobacter* may be compromised by inappropriate specimen collection or preparation procedures (*24*) or by antimicrobial therapy before stool collection. Deaths from undetected pathogens that cause gastroenteritis might consequently be attributed to gastroenteritis of unknown etiology. The UNEX project found that 15% of deaths that were initially unexplained by routine clinical testing were actually attributable to known pathogens, confirming that known pathogens are not always detected (*3*).

Noninfectious causes of gastroenteritis may also escape detection, notably drug-induced diarrhea, which has been associated with >700 different drugs, including antimicrobial agents (*25*). Diarrhea occurs in 2% to 25% of patients receiving antimicrobial drugs, depending on the drug (*26*). Approximately 10% to 20% of antimicrobial drug–associated diarrhea is attributable to opportunistic *Clostridium difficile* infections, but the etiologic agent in other cases is often unknown (*26*).

Physicians do not always recognize that many drugs besides antimicrobial agents can also cause diarrhea (*27*). Identifying other drugs as the cause of diarrhea may be difficult when a delay occurs between the start of drug therapy and the onset of diarrhea (*25*) or when patients are taking many drugs concurrently.

Many deaths occur among patients with drug-induced diarrhea, who may be receiving medications for systemic infections or other serious conditions. For example, hospital patients with antimicrobial drug–associated diarrhea attributable to *C. difficile* infections have a death rate of 4% to 10% (*28*). If the cause of drug-induced diarrhea was undetermined at the time of death, physicians may list diarrhea on hospital charts or death certificates without specifying a cause. These conditions will be coded as gastroenteritis of unknown etiology in hospital discharge or mortality databases.

Other noninfectious causes of gastroenteritis may not be promptly diagnosed, notably celiac disease, an inherited autoimmune disorder that can cause chronic diarrhea. Celiac disease may be as prevalent in the United States as in Europe, where it affects 0.3% to 0.4% of the population (*29*). On average, affected U.S. adults first received a diagnosis at age 45 after 11 years of symptoms that included diarrhea in 85% of patients (*29*). The delay in diagnosis is noteworthy because some persons are likely to die from other causes before celiac disease is recognized. Based on the average age at onset and diagnosis, the prevalence rate in Europe, and current U.S. death rates, approximately 250–325 adults with diarrhea attributable to undiagnosed celiac disease are expected to die each year. If diarrhea from undiagnosed celiac disease is reported on hospital charts or death certificates, it will be coded as gastroenteritis of unknown etiology.

Even when known causes of gastroenteritis are recognized, they may not be accurately coded in hospital discharge or mortality databases. Errors in hospital diagnosis coding often result from incomplete descriptions of diagnoses on the face sheet of medical records (*30*). Errors in mortality coding are frequently due to inaccurate completion of the cause-of-death section on death certificates (*31*). Recent studies indicate that 37%–41% of death certificates had improperly completed cause-of-death statements, including 18%–22% that listed nonspecific pathologic processes such as pulmonary edema without identifying the specific cause (*31**,**32*). The high frequency of this type of error suggests that some diagnosed causes of gastroenteritis are erroneously reported on death certificates when the nonspecific process (gastroenteritis) is listed without mentioning the specific cause and then coded as gastroenteritis of unknown etiology. However, the accuracy of gastroenteritis coding in hospital discharge and mortality databases does not appear to have been investigated.

Some evidence suggests that infectious causes of gastroenteritis are underreported on death certificates, presumably because the cause was either not detected or not recorded. Table 3 compares the average annual number of reported deaths from enteric infections that cause gastroenteritis with the number estimated by Mead et al. (*1*) from other sources. An estimated 1,333 deaths occurred, but only 110 (95% CI = 90–131) were reported. The discrepancy implies that approximately 1,200 annual fatal infections were not reported, including 500 *Salmonella* infections, 300 *Norovirus* infections, 100 enterohemorrhagic *E. coli* infections, and 100 *Campylobacter* infections. Some of the unreported infections may have been coded as gastroenteritis of unknown etiology, depending on how the infections were described on death certificates.

**Table 3:** Estimated and reported annual deaths from enteric infections that cause gastroenteritis, United States

Cause (ICD-10 code)	Estimated annual deaths^a^	Average annual reported deaths, 1999–2000 (95% CI)^b^
*Salmonella*, nontyphoidal (A02.0-A02.9)	582	59.0 (44.9–76.1)
*Noroviru*s (A08.1)	310	0.0 (0.0–3.7)
*Campylobacter* spp. (A04.5)	124	4.0 (1.1–10.2)
Enterohemorrhagic *Escherichia coli* (A04.3)	91	0.0 (0.0–3.7)
*Shigella* spp. (A03.0–A03.9)	70	11.5 (6.2–19.7)
*Cryptosporidium parvum* (A07.2)	66	12.5 (6.9–21.0)
Rotavirus (A08.0)	30	6.0 (2.2–13.1)
*Vibrio parahemolyticus* (A05.3)	20	1.5 (0.2–5.6)
*Brucella* spp. (A023.0–A023.9)	11	1.5 (0.2–5.6)
*Giardia lamblia* (A07.1)	10	2.0 (0.2–7.2)
*Clostridium perfringens* (A05.2)	7	4.5 (1.6–10.2)
Botulism, foodborne (A05.1)	4	4.5 (1.6–10.2)
*Salmonella typhi* (A01.0)	3	1.5 (0.2–5.6)
*Yersinia enterocolitica* (A04.6)	3	0.5 (0.0–3.7)
*Staphylococcus* food poisoning (A05.0)	2	1.5 (0.2–5.6)
Total	1,333	110.5 (89.9–131.3)

^a^Estimates from Mead et al. (*1*). ^b^Deaths with any mention of specified cause on the 1999–2000 Multiple Cause of Death files. Causes of death on the 1999–2000 files were coded by using ICD-10, which provides more detailed codes for enteric infections than ICD-9. The 95% confidence interval (CI) measures random variation in the number of deaths, which was assumed to follow a Poisson distribution for <100 deaths and a binomial distribution for >100 deaths.

## Deaths from Gastroenteritis of Unknown Cause

Some cases of gastroenteritis attributable to known causes are likely to be erroneously coded as gastroenteritis of unknown etiology in hospital discharge and mortality databases. Therefore, this study examined deaths attributed to gastroenteritis of unknown cause to determine whether any of the deaths may have involved known causes of gastroenteritis. Data were obtained from the 1994–1998 multiple-cause files, which provide more comprehensive and reliable estimates than the NHDS, using the modified ICD-9 definition of gastroenteritis of unknown etiology (Table 2). Data from more recent years were not examined because the ICD-10 codes used on later files are not fully comparable with the ICD-9 codes used on earlier files (*33*).

The average annual number of deaths attributed to gastroenteritis of unknown cause from 1994 to 1998 was 4,383 (Table 4), 12% lower than the estimate by the Mead study, which relied in part on NHDS data and included deaths from radiation, toxic, and drug-induced gastroenteritis. The characteristics of deaths attributed to gastroenteritis of unknown cause have been described in detail elsewhere (*21*). Characteristics potentially indicative of known causes of gastroenteritis include certain other causes of death and the large number of deaths in nursing homes.

**Table 4:** Deaths attributed to gastroenteritis of unknown cause, United States, 1994–1998

Characteristic	Average annual no. of deaths (%)^a^
Total	4,383 (100.0)
Selected other causes of death reported on death certificate (ICD-9 code)	
Enteric infectious diseases (001.0–008.8)	58 (1.3)
Septicemia of known etiology (038.0–038.8)	81 (1.8)
Bacterial pneumonia (481–482.9)	28 (0.6)
Septicemia of unknown etiology (038.9)	726 (16.6)
Hemorrhage of gastrointestinal tract, unspecified (578.9)	167 (3.8)
Acute or unspecified vascular insufficiency of intestine (557.0, 557.9)	136 (3.1)
Anemia, unspecified (285.9)	183 (4.2)
Acute or unspecified renal failure (584.9, 586)	443 (10.1)
Pneumonia of unknown etiology (485, 486)	463 (10.6)
Place of death	
Medical facility	3,058 (69.8)
Nursing home	766 (17.5)
Residence	476 (10.9)
Other, unknown	83 (1.9)

^a^Deaths with any mention of specified cause on the1994–1998 Multiple Cause of Death files.

Several causes of death reported on death certificates in conjunction with gastroenteritis of unknown cause suggest a possible cause for gastroenteritis. Approximately 1% of deaths had an ICD-9 code for an enteric infectious disease, which implies that physicians had erroneously listed gastroenteritis as a separate cause of death (Table 4). Similar proportions of deaths had codes for bacterial pneumonia or septicemia of known cause, conditions usually treated with antimicrobial drugs. These deaths may have involved simultaneous infections by known and unknown agents, but a more likely explanation is that antimicrobial drug–associated diarrhea occurred and was reported without specifying the cause.

Other causes of death might be indicative of fatal infections by known pathogens that were not accurately diagnosed or reported. Nearly 17% of deaths attributed to gastroenteritis of unknown cause were accompanied by septicemia of unknown cause (Table 4). Some of these deaths may have involved unreported *Salmonella* infections because this agent can also invade the bloodstream. Approximately 19% of deaths were associated with one or more conditions that could have been caused by unreported infections by Shiga toxin–producing pathogens such as the enterohemorrhagic *E. coli*. These conditions included gastrointestinal hemorrhage and vascular insufficiency of the intestine, which resemble hemorrhagic colitis, and renal failure and anemia, both elements of the hemolytic uremic syndrome. Nearly 11% of deaths involved pneumonia of unknown cause and may have included some unreported cases of Legionnaires' disease, which often involves diarrhea. Legionnaires' disease causes an estimated 800–2,700 deaths per year (*34*), but only 110 deaths per year from this condition were reported on death certificates from 1999 to 2000.

Nursing home residents accounted for 17.5% of the deaths from gastroenteritis of unknown cause from 1994 to 1998 (Table 4) and had the highest death rate from this cause of any group (*21*). The nursing home population is at risk from known enteric pathogens and drug-induced diarrhea, which possibly raises the death rate from gastroenteritis of unknown cause because these causes of gastroenteritis are not always recognized or reported. Outbreaks of infectious gastroenteritis are common in nursing homes, where the transmission of enteric pathogens among susceptible persons is facilitated by crowding, shared sources of food and water, and high rates of fecal incontinence (*35*). Although the cause of these outbreaks has often been undetermined, recent evidence suggests that the causal agents were usually noroviruses (*36*). Nonepidemic gastroenteritis in nursing homes is thought to be attributable primarily to noninfectious causes, including medications (*35*). Nursing home residents received an average of 6.7 concurrent medications each (*37*), which raises their risk for drug-induced diarrhea. Gastrointestinal symptoms (excluding bleeding) were the third most common type of adverse drug event in Massachusetts nursing homes (*38*) and occurred at an annual rate equivalent to 42,000 adverse events in the U.S. nursing home population.

## Discussion

No direct evidence indicates that unknown agents transmitted by food are a major cause of premature death in the United States. The lack of evidence is not surprising because most microorganisms resist cultivation on artificial media, and pathogenic agents that are difficult to culture have undoubtedly eluded identification (*39*). The innovative study of foodborne disease by Mead et al. (*1*) has increased awareness of the effects of unknown foodborne agents on health. However, their estimate of deaths from unknown foodborne agents depended on accurately estimating deaths from gastroenteritis of unknown cause, a category assumed to include all deaths from unknown foodborne agents. In fact, some unknown foodborne agents do not cause gastroenteritis, and some deaths attributed to gastroenteritis of unknown cause probably involved known causes of gastroenteritis that were either not detected or not reported, including enteric infections, adverse drug reactions, and celiac disease. The estimate of deaths from unknown foodborne agents consequently omitted deaths from unknown foodborne agents that do not cause gastroenteritis and almost certainly overstated the number of deaths from unknown foodborne agents that cause gastroenteritis.

Indirect estimates of deaths caused by unknown foodborne agents based on U.S. hospital discharge records or mortality data like the Mead study are inherently uncertain. Deaths due to unknown agents that cause gastroenteritis cannot be reliably distinguished from deaths due to known causes of gastroenteritis that were not accurately diagnosed or reported. Death certificate data suggest that many deaths attributed to gastroenteritis of unknown cause could have been due to known causes of gastroenteritis such as *Salmonella* infections or drug-induced diarrhea.

The number of deaths from unknown foodborne agents that do not cause gastroenteritis cannot even be indirectly estimated without first determining the clinical characteristics of such deaths. If the clinical characteristics of deaths caused by unknown foodborne agents are analogous, then one-half of the deaths caused by unknown foodborne agents do not involve gastroenteritis. However, this analogy may not be accurate because two of the known pathogens that cause fatal illnesses unaccompanied by gastroenteritis (*Toxoplasma* and *Listeria*) each cause substantially more deaths than the average foodborne pathogen and might be similarly unrepresentative of unknown foodborne agents.

More precise estimates of deaths attributable to unknown agents are likely to result from the ongoing UNEX project (*3*). Identifying the causal agents and their mode of transmission is the next step. Careful studies using molecular diagnostic techniques as well as epidemiologic methods might be able to determine whether unexplained deaths are associated with previously unknown microorganisms found in certain foods.

An improved estimate of the number of deaths caused by unknown agents transmitted by food could influence food safety policy in the United States. Regulatory decisions by the federal government are increasingly guided by economic cost-benefit analyses, which take into account the value of lives saved by preventing premature deaths. Although no consensus exists on how to value a life, the U.S. Food and Drug Administration currently employs a value of $5 million per life, a figure derived from wage studies (*40*). Using this value and the death estimate from the Mead study, the annual cost of deaths caused by unknown foodborne agents would be $17 billion. The actual cost of deaths caused by unknown foodborne agents is uncertain because the number of such deaths is not accurately known. The cost of effective measures to prevent deaths from unknown foodborne agents is similarly undetermined. Despite the uncertainty about the benefits and costs of reducing deaths from unknown foodborne agents, the possible economic losses from such deaths are so large that increased efforts to identify unknown foodborne agents appear to be warranted.
